# Development of Anodic Titania Nanotubes for Application in High Sensitivity Amperometric Glucose and Uric Acid Biosensors

**DOI:** 10.3390/s131014161

**Published:** 2013-10-21

**Authors:** Hsiang-Ching Lee, Li-Fan Zhang, Jyh-Ling Lin, Yuan-Lung Chin, Tai-Ping Sun

**Affiliations:** 1 Department of Mechatronic Engineering, Huafan University, Shihding, Taipei Hsien 223-01, Taiwan; E-Mail: fine@cc.hfu.edu.tw; 2 Department of Electronic Engineering, Huafan University, Shihding, Taipei Hsien 223-01, Taiwan; E-Mails: Zerzsr@hotmail.com (L.-F.Z.); chingyl888@gmail.com (Y.-L.C.); 3 Department of Electrical Engineering, National Chi Nan University, Puli, Nantou Hsien, 545-61, Taiwan; E-Mail: tps@ncnu.edu.tw

**Keywords:** titania nanotubes (TNTs), amperometry, glucose, uric acid (UA)

## Abstract

The purpose of this study was to develop novel nanoscale biosensors using titania nanotubes (TNTs) made by anodization. Titania nanotubes were produced on pure titanium sheets by anodization at room temperature. In this research, the electrolyte composition ethylene glycol 250 mL/NH_4_F 1.5 g/DI water 20 mL was found to produce the best titania nanotubes array films for application in amperometric biosensors. The amperometric results exhibit an excellent linearity for uric acid (UA) concentrations in the range between 2 and 14 mg/dL, with 23.3 (μA·cm^−2^)·(mg/dL)^−1^ UA sensitivity, and a correlation coefficient of 0.993. The glucose biosensor presented a good linear relationship in the lower glucose concentration range between 50 and 125 mg/dL, and the corresponding sensitivity was approximately 249.6 (μA·cm^−2^)·(100 mg/dL)^−1^ glucose, with a correlation coefficient of 0.973.

## 1. Introduction

Since their introduction in 1950, biosensors have undergone continuous development, and become widely used in industrial process control, food industries, environmental monitoring, and various applications in medicine and biotechnology. They are able to detect a small amount of enzyme on test paper. Formal development began in 1962, when Clark and Lyons proposed the “enzyme electrode”, which reacted dissolved oxygen (O_2_) with glucose, allowing the glucose concentration to be measured by monitoring the consumption of O_2_ [1]. Updike and Hicks fixed glucose oxidase with polyacylamide gel. The electrode was used to detect the dissolved oxygen to measure the glucose concentration as a first generation biosensor [2]. In 1974, Nanjo and Guilbault fabricated a platinum electrode which was covered with an immobilized uricase as a uric acid biosensor. Using a direct amperometric measurement, they were able to observe the decrease in the dissolved oxygen level by the uricase-catalyzed oxidation of uric acid [3]. In 1988 the Medisense Company used first-generation biosensors as the foundation of their research and development. A successful biosensor was developed using a mediator molecule. The sensitivity and speed of testing increased over time, and the pen type (Pen 2) and the credit card type (Companion) sensors were developed.

Titanium dioxide (TiO_2_, titania) has attracted great interest since the discovery of its photosensitivity [4], and due to its strong photo-oxidizing potential, high chemical stability, non-toxicity and a wide band gap semiconductor [5], so there are many applications for this transition-metal oxide, including use in pH sensors, gas sensors, biosensors, humidity sensors, radiation sensors, and solar cells [6–10]. In earlier work, we reported on TiO_2_ film with porosity that can be used to detect pH, sodium ions, potassium ions, and glucose by extended-gate ion-sensitive field effect transistors (EGFETs) and amperometric structures [11,12]. In the last decade, nanostructural materials have attracted increased scientific and technological attention due to their unique properties of high aspect ratio, large current-carrying capacity, good thermal stability, good mechanical strength and their potential applications.

Titania nanotubes (TNTs) have improved properties compared to any other form of titania for application in water and air purification photocatalysis, sensing, water photoelectrolysis for hydrogen generation, photovoltaics, photoelectrochemical solar cells, electronics, optics, tissue engineering and molecular filtration [13–18]. Titanium dioxide nanotubes and nanotube arrays have been produced by a variety of methods, including deposition into a nanoporous alumina template [19–22], sol–gel transcription using organo-gelators as templates [23,24], seeded growth [25], and hydrothermal processes [26–28]. However, of all nanotube fabrication methods, the architecture demonstrating by far the most remarkable properties are highly ordered nanotube arrays made by the anodization of titanium in fluoride-based baths [29–33], the dimensions of which can be precisely controlled. In 1991, Zwilling and co-workers [34] reported the porous surface of titania films electrochemically formed in fluorinated electrolyte by anodization. A decade later, in 2001, the fabrication of titanium dioxide nanotube arrays via anodic oxidation of titanium foil in a fluoride-based solution was first reported by Gong, *et al.* [14]. Further studies focused on the precise control and extension of the nanotube morphology, length and pore size, and wall thickness. Recently more research efforts have been devoted to the synthesis, characterization and applications of self-organized titanium dioxide nanotube arrays with well-defined and controlled nanostructures by means of acidic anodization of titanium foils [31,35]. The diameters and lengths of the titanium dioxide nanotube are easily tailored by controlling the synthesis parameters of the anodization process. Such ordered titanium dioxide nanotube arrays offer high surface areas, well defined nanostructures, favorable transport pathways, n-type properties, good adhesion to the substrate, and have thus become a popular choice of electrodes for chemical and biosensors, in which the sensitivity is dependent on both surface reaction and charge transfer.

Biosensors have developed rapidly over the last few years. Some clinically important analytes include glucose, uric acid (UA), *etc.* The analysis of biologically important analytes is the primary basis of disease diagnosis by clinicians. Therefore, the detection capabilities of small, portable biosensors are of particular importance in the medical field, and such measurement systems as the one used in this paper can be miniaturized, made portable, and thereby offer a significant advantage in on-site analysis.

## 2. Experiment

### 2.1. Anodization for Preparing TNT Array Films

**Figure 1.**
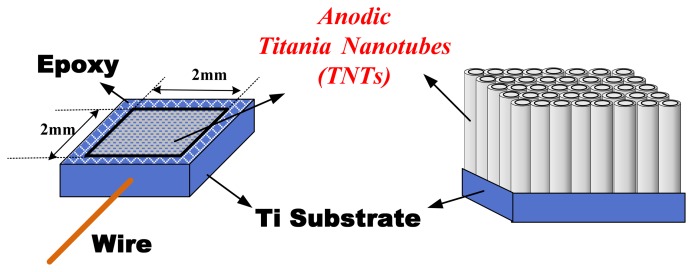
Planar view and cross section of the anodic titania nanotube array film electrode structure.

Titania nanotube array films were fabricated from high purity titanium foils (99.99%) by titanium anodization. First, the Ti foil was cleaned to remove the oil and then dried in an oven. Second, the Ti foil was formed by epoxy and connected with conductive wire as an anode-electrode, with a 2 mm × 2 mm electrode area covered by insulating epoxy. Third, the anode-electrode was placed in an electrolyte that consisted of ammonium fluoride, ethylene glycol and high-purity de-ionized water (DI water) [17,18]; only two different ratios of these components were used in this study. The compositions of these two types of electrolyte are shown in Table 1, where electrolyte baths A and B have different H_2_O contents. Finally, the TNTs array films were fabricated at room temperature using a platinum cathode-electrode and potentiostatic mode at 60 V for 1 h. The TNTs array film electrode structure is illustrated in Figure 1.

The formation of nanotubes in an electrolyte that contains hydrogen ions (H^+^), oxygen anions (O^2−^) and fluorine anions (F^−^) is described by a localized dissolution model [29], with an oxide layer initially formed due to interaction of the surface titanium ions (Ti^4+^) with oxygen ions in the electrolyte, and via the following reactions [36]:

(1) $$2{\text{H}}_{2}\text{O}\to {\text{O}}_{2}+4{\text{H}}^{+}+4{\text{e}}^{-}$$

(2) $${\text{Ti}}^{4+}+{\text{O}}_{2}\to {\text{TiO}}_{2}$$

The pore formation occurs as a result of the localized chemical dissolution of the oxide by F^−^, according to the following reaction [36]:

(3) $${\text{TiO}}_{2}+{6\text{F}}^{-}+{4\text{H}}^{+}\to {\text{TiF}}_{6}^{2-}+2{\text{H}}_{2}\text{O}+{4\text{H}}^{+}+{4\text{e}}^{-}$$

This leads to a higher field at the bottom of the pore that drives further oxidation and field assisted dissolution, where titanium ions come out of the metal and dissolve in solution. The metallic region between the pores also undergoes a similar transition, leading to tube formation. For a given rate of pore formation, the chemical dissolution of the oxide at the pore mouth by fluorine anions determines the tube length. Higher anodization voltages increase the oxidation and field-assisted dissolution; hence, a greater nanotube layer thickness can be achieved before equilibrating and then chemical dissolution [15,37].

### 2.2. Preparation of Uric Acid and Glucose Biosensors on TNT Array Films

Glucose and uric acid biosensors were manufactured using glucose oxidase (GOD) and urate oxidase (uricase), and covering the TNTs array films with ferrocenecarboxylic acid with PVA-SbQ, using the entrapment method. Polyvinylalcohol-bearing styrylpyridinium groups (PVA-SbQ: degree of polymerization 3500, degree of saponification 88, betaine SbQ 1.05 mol%, solid content 10.22 mol%, pH 5.7 and SPP-H-13) were obtained from Toyo Gosei Kogyo (Chuo-Ku, Tokyo, Japan). GOD and uricase were both purchased from Sigma. All reagents were of analytical grade. Glucose and uric acid biosensors were prepared as follows [11]:

Step 1. First, glucose oxidase (9 mg) or urate oxidase (10 mg) and luxuriant irons (ferrocenecarboxylic acid) were placed in a phosphate buffer liquid solution (phosphate-KOH buffer solution, PBS, 100 μL). The concentration of the phosphate buffer liquid solution was 5 mM and its pH was 7.0.
Step 2. PVA-SbQ (100 mg) was placed in a phosphate buffer liquid (100 μL). They were mixed in a 1:1 proportion.
Step 3. Oxidized enzyme liquid (1 μL) and PVA-SbQ buffer mixture was dropped onto the TNTs sensing window. The whole was then exposed to an ultraviolet lamp for 20 min, and then stored under dry conditions for about 24 h at 4 °C.
Step 4. The above sample was soaked for 60 min in DI water, and then soaked in the Phosphate-KOH Buffer Solution (PBS, 5 mM, pH 7.0) for about 1 h before use.

### 2.3. The Cyclic Voltammetry Measurement System

Uric acid and glucose measurement responses were based on the electrochemical oxidation of H_2_O_2_. The oxygen atoms serve as the electron transduction mediators, which catalyze the production of H_2_O_2_ as byproducts of UA and glucose. The hydrogen peroxide is electrochemically oxidized at the sensing electrode. Therefore, the UA and glucose concentration can be determined by measuring the current in the sensing electrode. However, oxygen is extremely active, and the oxidation or reduction over-potential of H_2_O_2_ on an electrode surface is large, and electronically active components are generated that interfere with the reaction. Therefore, a small electron transduction mediator molecule, ferrocene, must be added to the enzymatic electrode to catalyze the reduction of H_2_O_2_, and to reduce the reduction over-potential of H_2_O_2_ on the electrode surface [38]. The oxidases catalyze the oxidation of substrates according to the follow reactions [39]:

(4) $$\begin{array}{l} \text{Uric acid}+{\text{O}}_{2}+2{\text{H}}_{2}\text{O}\overset{\text{Uricase}}{⟶}\text{Allantion}+\text{C}{\text{O}}_{2}+{\text{H}}_{2}{\text{O}}_{2}\\ {\text{H}}_{2}{\text{O}}_{2}+{2\text{H}}^{+}+{2\text{e}}^{-}\overset{\text{Ferrocene}}{⟶}2{\text{H}}_{2}\text{O} \end{array}$$

(5) $$\begin{array}{l} \text{Glucose}+{\text{O}}_{2}+2{\text{H}}_{2}\text{O}\overset{\text{GOD}}{⟶}\text{Gluconic acid}+\text{C}{\text{O}}_{2}+{\text{H}}_{2}{\text{O}}_{2}\\ {\text{H}}_{2}{\text{O}}_{2}+{2\text{H}}^{+}+{2\text{e}}^{-}\overset{\text{Ferrocene}}{⟶}2{\text{H}}_{2}\text{O} \end{array}$$

The cyclic voltammetry (CV) method is a type of oxidation-reduction reaction measurement. The system includes a biosensor as a working electrode, and a reference electrode (RE) using Ag/AgCl forming a two-electrode setup. The potential was cycled between −3 V and 3 V at a scan rate of 120 mV/s.

### 2.4. The Amperometry Measurement System

**Figure 2.**
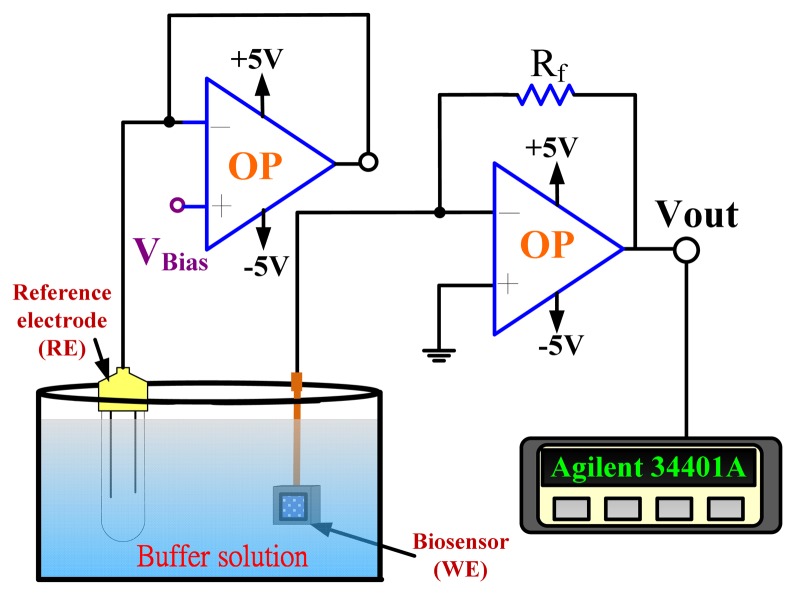
The amperometry measurement system.

An amperometric readout circuit (Figure 2) including two operational amplifiers and a 10 kΩ resistor (R_f_) was used for biosensor measurement.

The amperometric measurement system used biosensors as the working electrode, and the Ag/AgCl reference electrode (RE) which delivered the base potential for the working electrode. The output current density was:

(6) $${\text{J}}_{\text{biosensor}}=\frac{{\text{I}}_{\text{biosensor}}}{{\text{A}}_{\text{rea}}}=\frac{{\text{V}}_{\text{out}}}{{\text{A}}_{\text{rea}}\cdot {\text{R}}_{\text{f}}}=\frac{{\text{V}}_{\text{out}}}{0.04\cdot 10k}=2500\cdot {\text{V}}_{\text{out}}(\mu A/cm^{2})$$

where A_rea_ is the area of electrode with 2 mm × 2 mm.

## 3. Results and Discussion

### 3.1. TNT Array Films

#### 3.1.1. Titanium Anodization

**Figure 3.**
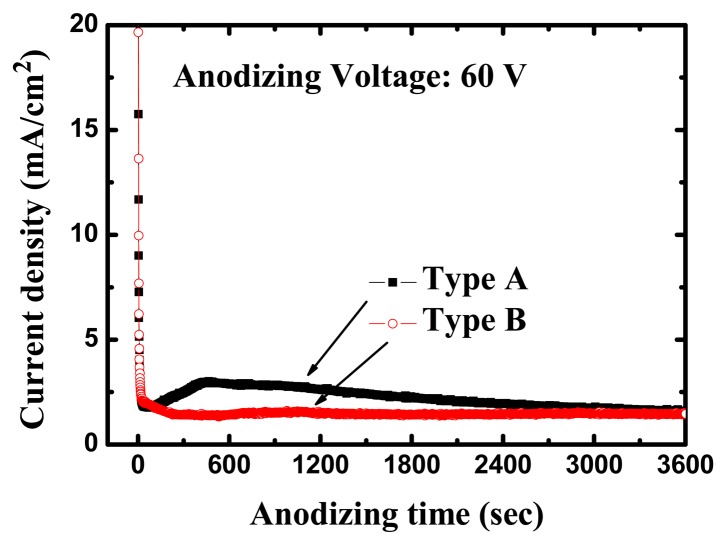
The variation of current density in anodization process for different electrolytes.

The high-purity (99.99%) titanium foils used in this work were obtained from Alfa Aesar (Ward Hill, MA, USA). All anodization experiments were conducted at room temperature (24 °C). The anodizing voltages were kept at 60 V for 1 h with a platinum counter-electrode during the entire process. Figure 3 shows the variation of current density during the anodization process for different electrolytes. Within 1 min after application of the anodizing voltage, the measured current density was reduced from >30 mA/cm^2^ to a local-minimum of 1.77 mA/cm^2^; about 400 sec later, it reached a maximum of approximately 3 mA/cm^2^, after which the current density gradually decreased to 1.55 mA/cm^2^ for the type A electrolyte with 20 g less water. In the first 60 s of the anodization the current density was observed to drop drastically to 1.96 mA/cm^2^, and afterwards remained stable at around 1.4 mA/cm^2^ for the type B electrolyte with 50 g more water. With reference to the anodization behavior, the current density curve for the type A electrolyte was more active than that of the type B electrolyte in our experiment.

#### 3.1.2. Surface Analysis

**Figure 4.**
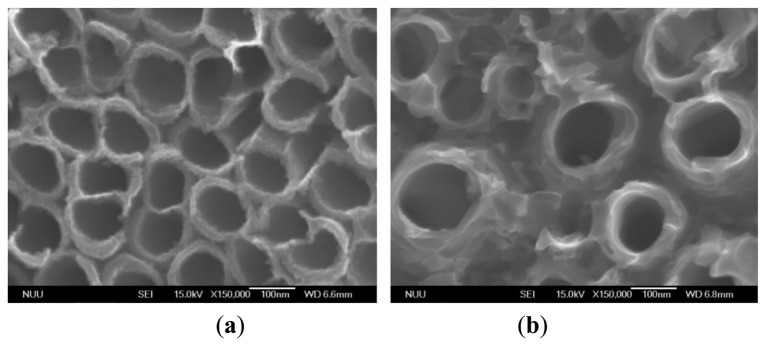
FE-SEM images of titania nanotubes grown by electrochemical anodization in (**a**) Type A electrolyte, and (**b**) Type B electrolyte.

**Figure 5.**
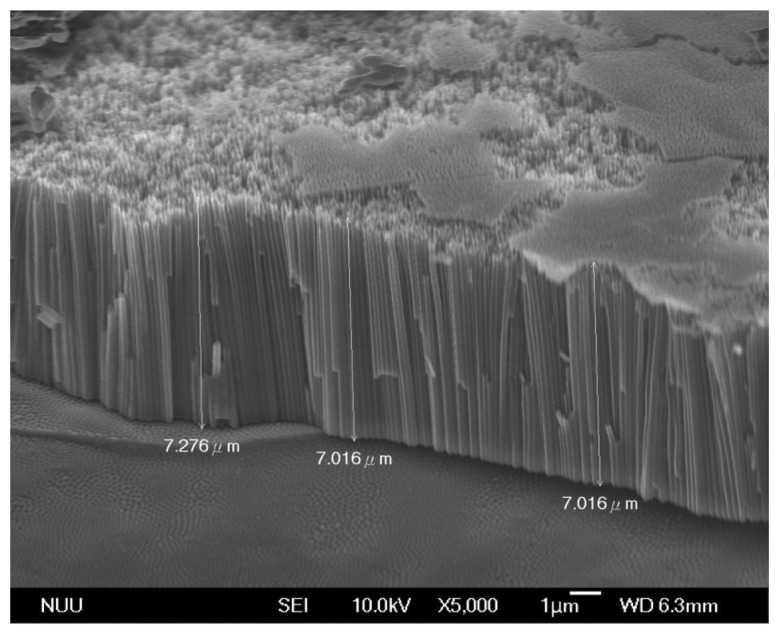
A FE-SEM cross-sectional image of titania nanotubes made by anodization grown at 60 V in an ethylene glycol solution for 1 h.

The composition of the electrolyte used is a critical factor for nanostructures during anodization. In this work, two types of TNTs array films were obtained by different electrolytes, and the corresponding NH_4_F concentration of electrolyte type A is 0.05 wt% greater than that of type B. Figure 4 shows the field emission scanning electron microscope (FE-SEM) analysis for the type A TNTs array films (Figure 4a), and the type B TNTs array films (Figure 4b). The tube inner diameters averaged 101 nm and 117 nm, respectively, the ultrathin tube walls were about 20 nm and 30 nm thick for type A and type B TNTs array films, respectively. The tube lengths were approximately 7 μm as shown in Figure 5. According to the FE-SEMs, the architecture demonstrated that the quality of type A TNTs array films was uniformly high. In addition, the surface areas of type A TNTs array films were greater than those of type B. Therefore, we fabricated glucose and uric acid biosensors based only on the type A TNTs array films.

### 3.2. Uric Acid Biosensors with TNT Films

#### 3.2.1. Cyclic Voltammetry Analysis

**Figure 6.**
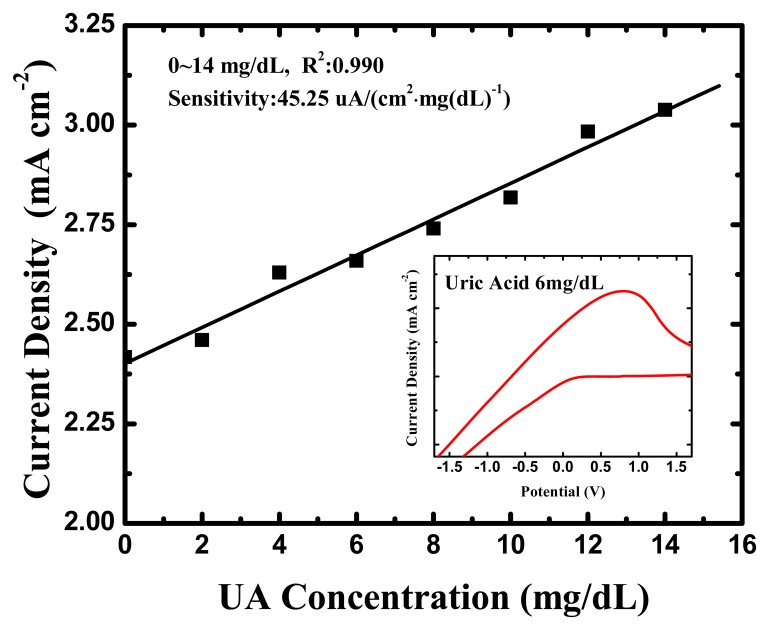
The CV measurement results of the uric acid biosensor.

The cyclic voltammetry measurement of uric acid cycled through the range of −3 V and 3 V potential at a scan rate of 120 mV/s. The inset of Figure 6 indicates that a sensitive anodic peak current occurred in the potential range between 0.8 V and 1 V at a UA concentration of 6 mg/dL. It can be inferred that TNTs are an efficient electron conducting tunnel facilitating electron transfer, and electrochemical oxidation was promoted. Figure 6 displays a typical plot of the anodic peak current *versus* UA concentration for a uric acid biosensor. The response peak current densities of the UA biosensor had a 45.25 (μA·cm^2^)·(mg/dL)^−1^ sensitivity, and a correlation coefficient of 0.990, between UA concentrations of 0 and 14 mg/dL.

#### 3.2.2. The Amperometry Measurement Systems

**Figure 7.**
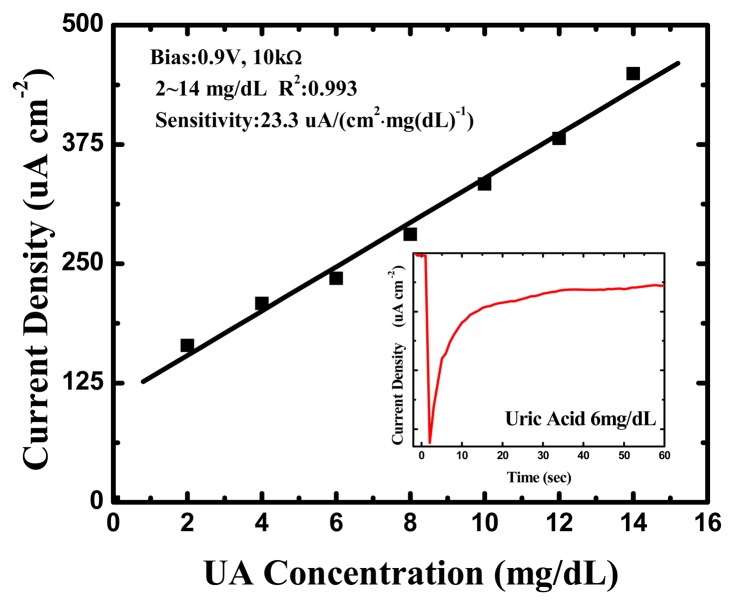
Uric acid concentration response of the uric acid biosensor with amperometric readout circuit.

The amperometric readout circuit shown in Figure 2 was used as the amperometry measurement system for each UA concentration (0, 2, 4, 6, 8, 10, 12 and 14 mg/dL) and the bias was set at 0.9 V. One of the original response curves by amperometric readout circuit at a UA concentration of 6 mg/dL is shown in the inset of Figure 7.

The response was very rapid, and an available current signal was generated within a few seconds in every UA concentration. Furthermore, they exhibited splendid linearity for UA concentrations between 2 and 14 mg/dL, and the sensitivity was approximately 23.3 (μA·cm^−2^)·(mg/dL)^−1^ UA, with a correlation coefficient of 0.993, as shown in Figure 7. This is higher than the UA biosensors based on other nanomaterials [40–44] and the biomimetic titanate nanotubes by hydrothermal decomposition [45], the corresponding detailed comparisons are shown in Table 2.

**Figure 8.**
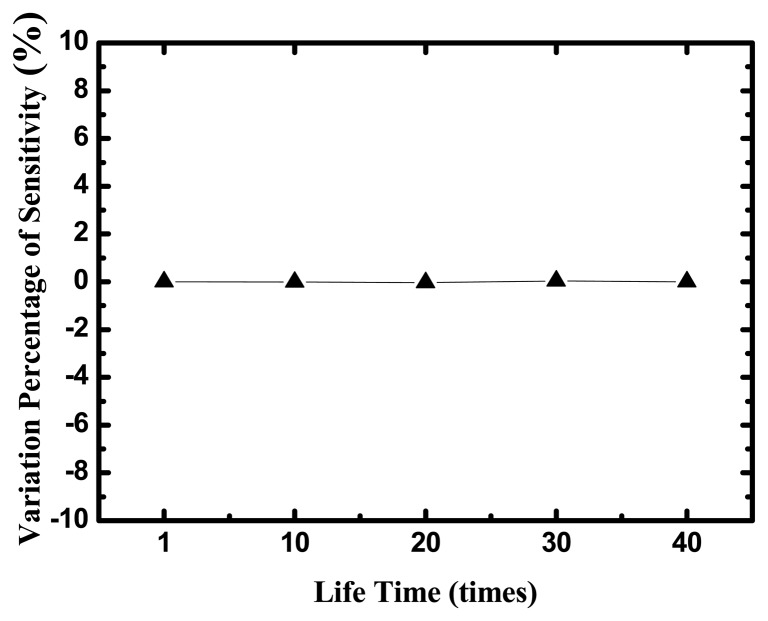
The life time of the uric acid biosensors on TNTs films.

The life time of the uric acid biosensor is investigated in Figure 8, where y-axis is the variation percentage of sensitivities and x-axis is the times of measurements during five days and eight tests every day.

The uric acid biosensors were stored under dry conditions at 4 °C in a refrigerator when they were not in use. After 40-times measurements in different UA concentrations, the variation percentage of sensitivities is small 5%.

### 3.3. Glucose Biosensors with TNT Films

**Figure 9.**
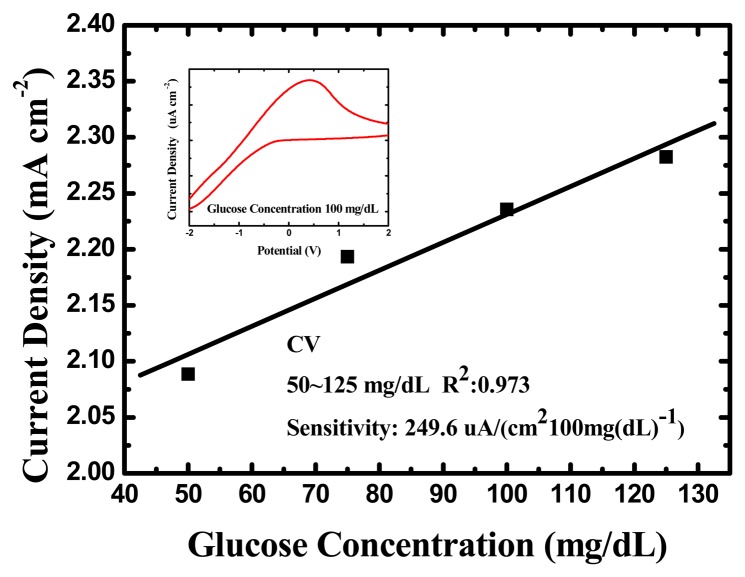
The CV measurement results of the glucose biosensor.

The glucose biosensor assayed with the cyclic voltammetry measurement as Section 3.2.1 presented a good linear relationship between current density and glucose concentration in the lower glucose concentration range between 50 and 125 mg/dL, and the corresponding sensitivity was approximately 249.6 (μA·cm^−2^)·(100 mg/dL)^−1^ glucose with a correlation coefficient of 0.973. The calibration curve of the glucose biosensor is plotted in Figure 9. The sensitivity demonstrates better than other literatures that based on nanostructured metal-oxides [46–49] except ZnO nanowire reported by Basu *et al.* and MWNTs/CuO nanoparticle reported by Jiang *et al.* [50]. Nanostructured metal-oxides have been extensively studied to develop biosensors with high sensitivity and fast response time for glucose by electrochemical oxidation. The results of our glucose biosensors manufactured on TNTs indeed prove the characteristics of nanomaterials.

**Figure 10.**
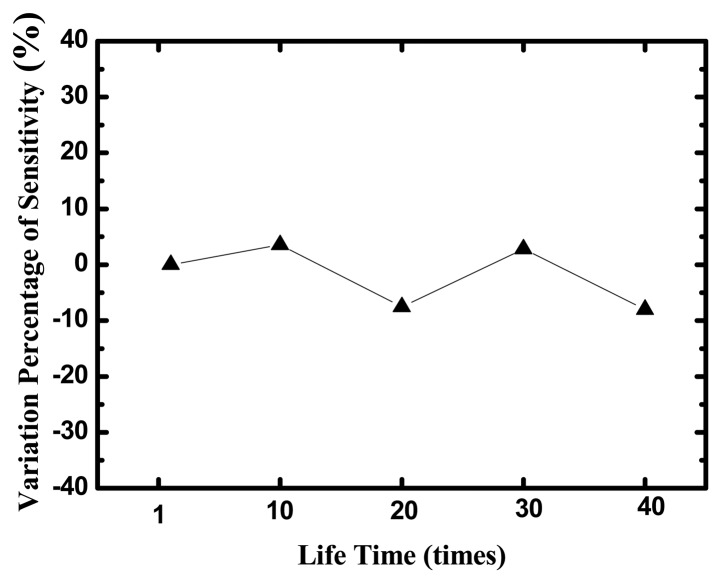
The life time of the glucose biosensors on TNTs films.

The life-time test of the glucose biosensor with TNTs films made by anodization is displayed in Figure 10. The sensors were measured during five days and eight tests were performed every day. After 40 measurements at different glucose concentrations, the variation percentage of sensitivities is small 10%.

## 4. Conclusions

In this study, we developed a novel nanoscale biosensor using titanium oxide nanotubes made by anodization. The anodization treatment created large surface areas on the tubes' inner and outer sidewalls, which were involved in redox reactions and formed a microenvironment favorable for enhancing the direct electron transfer between the enzymatic active sites and the electrode. Otherwise, some metal-oxide nanomaterials have high isoelectric point (IEP) and a good surface for oxidase immobilization. Therefore, much attention to these electrode materials will be paid. Since titanium oxide has high IEP (3.9–8.2) dependent on the manufacture method and is environmentally-friendly [50], we have frequently proposed it as a prospective interface for the immobilization of biomolecules. In this study, biosensors with TNTs for uric acid had an excellent linearity for UA concentrations by amperometry in the range between 2 and 14 mg/dL, with 23.3 (μA·cm^−2^)·(mg/dL)^−1^ UA sensitivity, namely 450 (μA·cm^−2^)·(mM)^−1^ UA, and a correlation coefficient of 0.993. On the other hand, the biosensors for glucose also had good linearity between 50 and 125 mg/dL glucose, and high sensitivity, 249.6 (μA·cm^−2^)·(mg/dL)^−1^ glucose, with a correlation coefficient of 0.973 by amperometry. Furthermore, it is easy to fabricate the TNTs for any kind of flexible and bendable material, even at room temperature.

## Figures and Tables

**Table 1.** The composition of electrolytes.

**Type**	**Ethylene Glycol (mL)**	**NH_4_F (g)**	**DI Water·(mL)**
A	250	1.5	20
B	250	1.5	50

**Table 2.** Comparion of some uric acid biosensors based on nanostructures.

**Group [reference]**	**sensitivity** ***μA/cm****^2^/****mM***	**Dynamic Range**	**Method**	**Manufacture**
Fenfen Zhang, *et al.* [40]	**105.4**	**0 μM∼15 μM**	**Nafion coating**	GCE/ZnO nanorods carboxylated
Xing-Jiu Huang, *et al.* [41]	**34.2**	**0 μM∼18μM**	**Surface adsorption-controlled**	SWCNT net electrode
P. Kannan, *et al.* [42]	**75**	**50 nM∼0.4μM**	**Self-assembly and seeding**	DMT/AuNPs
Yiting Wang, *et al.* [43]	**393**	**5 μM∼1 mM**	**Vapour Liquid Solid (VLS) growth.**	MWNTs/ZnO nanoparticles
Haisheng Tao, *et al.* [45]	**184.3**	**1 μM∼5 mM**	**Hydrothermal decomposition**	GCE/TONTs
Rachna Rawal, *et al.* [44]	**100**	**120 μM∼830 μM**	**Electrochemical** **deposition**	Au/PANI/cMWCNT/PBNPs
		**(5 μM∼830 μM)**		
**This study**	**394**	**120 μM ∼ 830 μM**	**Anodization**	Ti/ATONTs
		**(30 μM∼830 μM)**		
